# The Surface Deterioration of Prefabricated Zirconia Crowns on Exposure to Acidulated Phosphate Fluoride Gel: An In Vitro Study

**DOI:** 10.7759/cureus.66371

**Published:** 2024-08-07

**Authors:** Sanidhya L Makwana, Kalpesh Vaishnav, Ravi Joshi, Tulsi H Patel, Neil N Vora, Nishit H Sachde, Keyur A Vala, Zalak Raval, Jinsa A Yohannan, Vidhi J Joshi

**Affiliations:** 1 Department of Prosthodontics, Crown and Bridge, Karnavati School of Dentistry, Gandhinagar, IND; 2 Department of Dentistry, University of the Pacific, San Francisco, USA; 3 Department of Conservative Dentistry and Endodontics, Government Dental College and Hospital, Ahmedabad, IND; 4 Department of Paediatric and Preventive Dentistry, Karnavati School of Dentistry, Gandhinagar, IND

**Keywords:** topographical changes, fluoride, surface roughness of crowns, apf gel, fe-sem analysis, prefabricated zirconia crowns

## Abstract

Introduction

Zirconia is a widely used restorative material in dentistry due to its superior aesthetic and mechanical properties. The oral cavity is a complex ecosystem with various components, which affect the teeth, as well as artificial restorative materials. Various personal and professional interventions carried out can severely affect the properties of restorative materials, thus altering the longevity of the prosthesis; 1.23% acidulated phosphate fluoride (APF) gel is one such professionally applied topical fluoride agent used to prevent caries. The interaction of this APF gel with highly aesthetic restorative material such as zirconia crowns is unknown.

Objective

The objective of this study is the evaluation of the surface deterioration of prefabricated zirconia crowns on exposure to deionised water and 1.23% acidulated phosphate fluoride (APF) gel with field emission scanning electron microscope (FE-SEM) and mass loss analysis.

Material and method

Sixty prefabricated paediatric zirconia crowns were taken, 10 samples were immersed in deionised water, 40 samples were immersed in 1.23% APF gel and 10 samples were used as control. Surface morphology and mass loss analysis were carried out at time intervals of four minutes, 24 hours, 48 hours and 72 hours using FE-SEM and digital weighing machine.

Results

No visual change was observed in the samples immersed in deionised water at the time interval of 72 hours. There was a marked visual change in samples immersed in 1.23% APF gel at the time interval of four minutes to 72 hours; this change involved a loss of gloss to the appearance of chalkiness. FE-SEM analysis for the control group and samples immersed in deionised water showed a smooth, continuous, undisrupted top layer, while samples immersed in 1.23% APF gel showed changes ranging from surface etching, to pinhole porosities, to crack formation and disruption of the surface depending upon the exposure time.

Conclusions

On the immersion of zirconia crowns in an aqueous acidic medium of 1.23% APF gel, the crowns showed flaws, imperfections and uneven superficial layers. It has been observed that surface grains are disrupted and micropores have been formed. This degraded superficial surface when undergoes cyclic mechanical loading can accelerate the ageing phenomenon of zirconia. Mechanical forces along with a dynamic electrochemical environment can degrade the material properties of zirconia.

## Introduction

Zirconia and its alloys are gaining prevalence in the dental implant sector because of their aesthetic properties, biocompatibility, low plaque surface adhesion, high flexural strength and desirable osseointegration, absence of mucosal discolouration and corrosion resistance [1-3]. The ongoing research for aesthetic, functionally stable and biocompatible materials has favoured the use of all-ceramic reconstructions for fixed dental prostheses (FDPs) as alternatives to conventional porcelain-fused-to-metal (PFM) prostheses [4].

Recently, ceramic restorations have become progressively more popular, particularly those involving monolithic zirconia [5] frequently employed in the development of long-lasting fixed restorations [6,7] due to its aesthetic value and biocompatibility [8], as well as physical and structural characteristics [9-11].

Traditionally, subtractive manufacturing (SM) with computer-aided design and computer-aided manufacturing (CAD-CAM) has been adopted to create zirconia restorations [1]. Additive manufacturing (AM), also known as three-dimensional (3D) printing utilising various tools, has lately acquired popularity for the fabrication of dental ceramics, particularly zirconia [12].

Zirconia crowns were found to have a longer duration of survival and lower rates of complications in a prospective study with a 15-year follow-up [13]. Zirconia-based all-ceramics are currently used to fabricate copings, implant abutments and partial and complete arch frameworks on both natural teeth and implants, in both anterior and posterior oral cavity areas [14-17]. Despite the high success rate of zirconia as a prosthetic material, there have been case reports mentioning the catastrophic failure of zirconia prosthesis intraorally. The failure of the ceramic prosthesis is due to the brittleness of the material, a quality inherent to the material [2].

Polished monolithic zirconia maintains lower levels of enamel wear compared to metal ceramics, feldspathic porcelains and lithium disilicate. The opposing enamel wear induced by polished monolithic zirconia will be either equal to or less than that of natural enamel wear [18]. There is decreased tensile bond strength of zirconia crowns and resin nanoceramics after thermomechanical ageing [19].

The use of ultrasonic scaling led to a higher surface roughness on zirconia and lithium disilicate crowns tested, in comparison to both manual metal and plastic scaling methods. On all ceramics, plastic tips resulted in the lowest surface roughness, making them a preferable choice for all restorations, particularly for implant-supported ceramic restorations [20].

It has also been proposed that acidic aqueous conditions might expedite the process of ageing, with fracture formation lowering the lifetime of the prosthesis by 20%-30% [3]. The oral environment is an aqueous electrochemical medium with high odds of pH variations caused by the presence of bacteria, intake of meals or the presence of an inflammatory disease [21]. Furthermore, patients are generally prescribed topical oral hygiene mouthwashes that are acidic in nature, attributed to sodium fluoride and hydrochloric acid [22]. As a result, it is critical to study and comprehend the impact of an unfavourable acidic aqueous condition on the surface of zirconia used as a prosthetic material.

The impact of corrosive environments was investigated for different dental ceramics, where the decreased pH of the medium damaged the surface, culminating in rough surfaces [23]; 1.23% acidulated phosphate fluoride (APF) gel is a commonly applied topical fluoride used to prevent dental caries. Although the influences of different pH values on zirconia have been evaluated, the effects of topical fluoride agents, such as APF gel, on the surface of zirconia crown have not been investigated [24].

Hence, this study aims to understand and evaluate the surface characteristic deterioration of prefabricated zirconia on exposure to 1.23% APF gel and analyses with field emission scanning electron microscope (FE-SEM) and mass loss analysis. This study was based on the assumption that an acidic aqueous medium in the oral cavity could accelerate the ceramic ageing phenomenon. This process could lead to nucleation and crack propagation, which can fracture the material ultimately causing the dissolution of ceramic particles in the oral environment. The overall focus of this study was to understand the effect of the exposure of zirconia crowns to 1.23% APF gel.

Objectives

The objective of this study is to study the corrosion behaviour of prefabricated zirconia crowns on exposure to 1.23% APF gel and evaluate the surface deterioration of prefabricated zirconia on exposure to deionised water and 1.23% APF gel with field emission scanning electron microscope and mass loss analysis.

## Materials and methods

This study was conducted at the Department of Prosthodontics, Crown and Bridge, Karnavati School of Dentistry. No human subjects were involved in this study, so it did not require any ethical clearance.

Materials

Prefabricated paediatric zirconia crowns (Signature Crowns, Ahmedabad, Gujarat, India) deionised water (ACS Chemicals, Ahmedabad, Gujarat, India) and 1.23% APF gel (Fluorovil, Vishal Dentocare, Ahmedabad, Gujarat, India). Prefabricated zirconia crowns were selected for the study to maintain standardisation and avoid any errors that can occur during the custom fabrication of zirconia crowns.

Preparation of sample

The dimensions of all the samples were adjusted to be less than 10 × 10 × 4 mm to fit in the holding device of SEM. All the samples were immersed in acetone and air-dried for three minutes before immersion in test media.

Method

**Figure 1:**
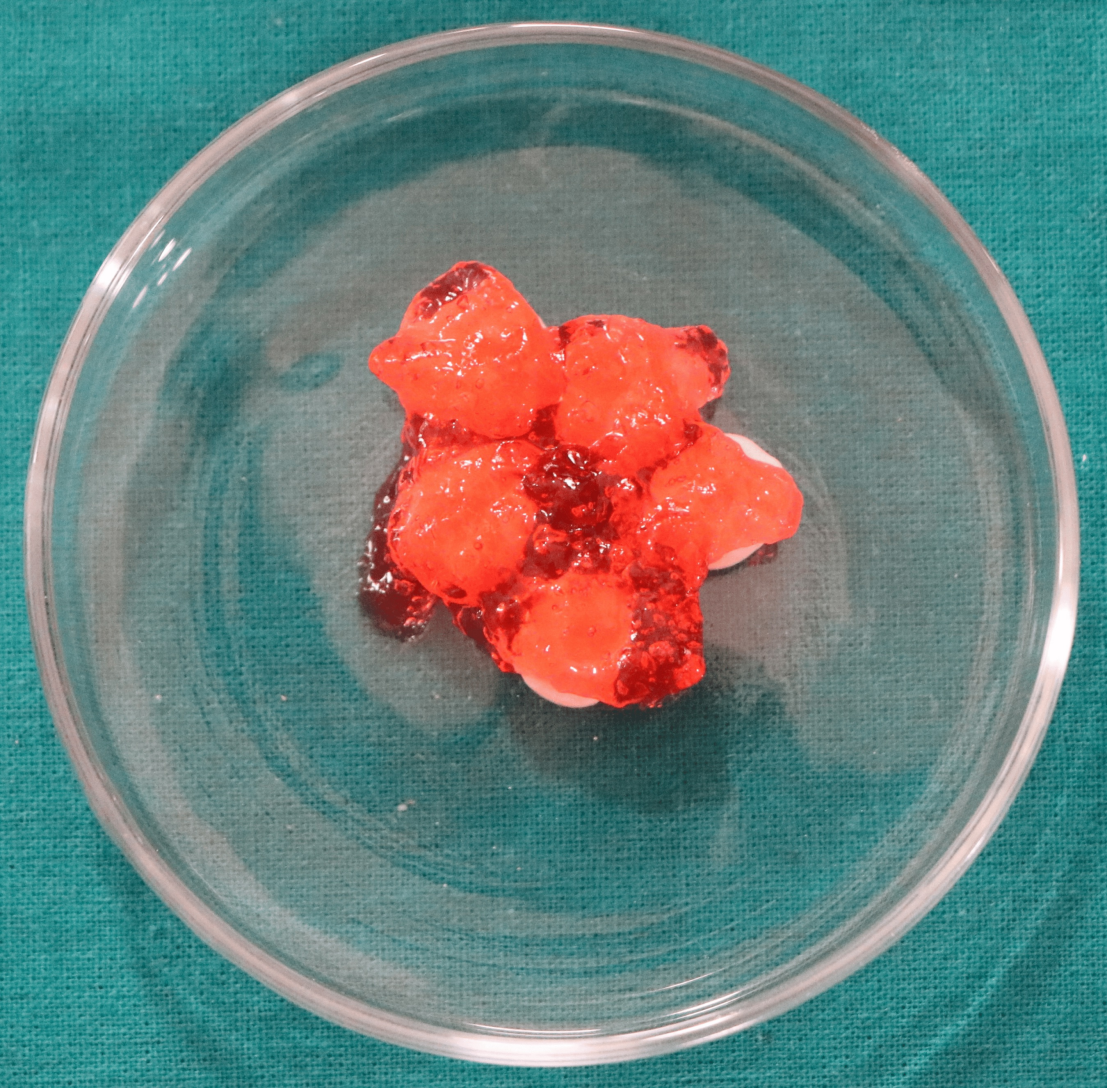
Prefabricated zirconia crowns immersed in 1.23% acidulated phosphate fluoride (APF) gel. Image credit: Dr. Sanidhya Makwana.

All sixty samples were divided into three groups according to different immersion conditions: group 1, 10 prefabricated zirconia crowns not immersed in any medium used as a control; group 2, 10 prefabricated zirconia crowns immersed in deionised water; group 3, 40 prefabricated zirconia crowns immersed in 1.23% APF gel (Figure 1); group 3.1, 10 samples immersed in 1.23% APF gel for four minutes; group 3.2, 10 samples immersed in 1.23% APF gel for 24 hours; group 3.3, 10 samples immersed in 1.23% APF gel for 48 hours; and group 3.4, 10 samples immersed in 1.23% APF gel for 72 hours.

The neutral pH oral environment for the material was represented by samples immersed in deionised water; 1.23% APF gel represented acidic oral environmental conditions that simulated the application of topical fluoride. In order to simulate oral temperature, all the test samples were stored at a 37°C room temperature. Twenty-four hours of immersion equates to one year of daily exposure to an APF solution for four minutes. Forty-eight hours of immersion represents four minutes of daily exposure for 1.5 years, and 72 hours of immersion represents four minutes of daily exposure for three years. 

Surface morphology and mass loss analysis of all the samples were evaluated at time intervals of four minutes, 24 hours, 48 hours and 72 hours.

According to American Society of Mechanical Engineers (ASME) guidelines, which states that the examination of the subject should be done within 600 mm distance from the naked eye and at an angle greater than 30 degrees [25], the direct visual examination of the samples without any aid was carried out. A standard light level of 500 lx was employed [26].

A field emission scanning electron microscope (FE-SEM) (JSM-7600F, JEOL, Akishima, Tokyo, Japan) was employed to topographically examine the surface morphology of all samples. Samples from all the groups were air-dried and coated with platinum to make them conductive for FE-SEM analysis. Mass loss analysis was performed using a digital weighing unit (Mettler Toledo ME204, Greifensee, Switzerland) to weigh the samples before and after immersion. This digital weighing machine could measure weight accurately up to 0.1 mg [27].

## Results

All the samples subjected to immersion in 1.23% APF gel showed visual and topographical changes, whereas no sample from the control group and those immersed in distilled water showed any change. So, there was no probability equation formation in the study. According to a study conducted by Trabelsi et al. [28] and Thomas et al. [29], our study can be categorized as an image-based study, which does not involve any numericals, and hence requires no statistical analysis.

Visual examination analysis

**Figure 2:**
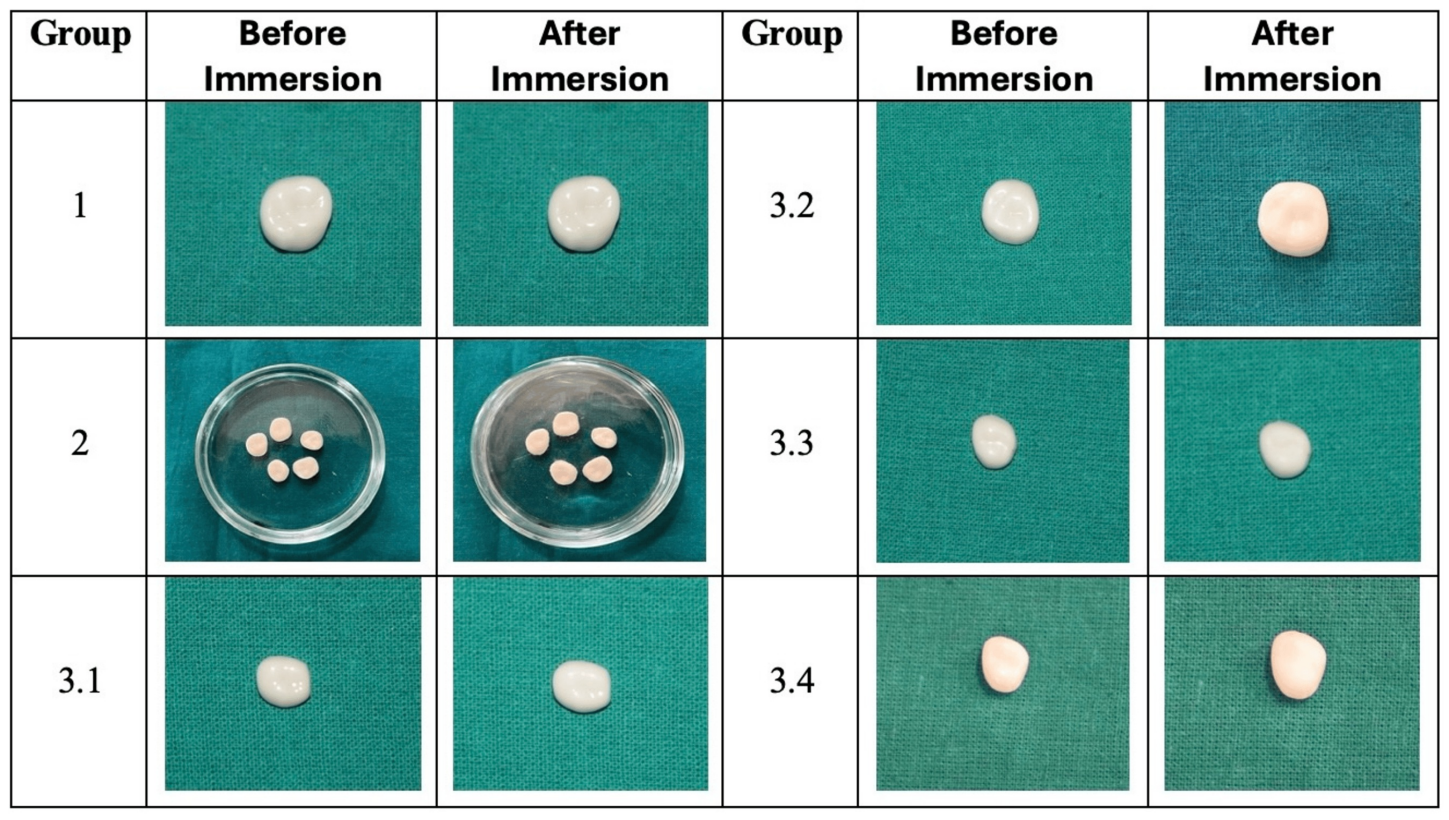
Images showing visual changes on the surface of zirconia crowns. Group 1 samples not immersed in any medium used as a control. Group 2 samples immersed in deionised water. Group 3.1 samples immersed in 1.23% APF gel for four minutes. Group 3.2 samples immersed in 1.23% APF gel for 24 hours. Group 3.3 samples immersed in 1.23% APF gel for 48 hours. Group 3.4 samples immersed in 1.23% APF gel for 72 hours. Image credit: Dr. Sanidhya Makwana. APF: acidulated phosphate fluoride

Visual changes in all the groups can be depicted in Figure 2.

A change in surface morphology was observed as a loss of glaze and/or chalky appearance upon direct visual inspection of all samples under standard conditions except the control group. Group 1 is the control sample that was not subjected to any immersion and showed no change in the surface morphology when visually analysed after 72 hours. Group 2 samples immersed in deionised water showed similar lustre and glaze as compared to the control sample after 72 hours of immersion. Group 3.1 samples were immersed in 1.23% APF gel for four minutes; a marked loss of glaze could be seen on visual comparison. Group 3.2 samples were immersed in 1.23% APF gel for 24 hours; a total loss of surface glaze was observed. Group 3.3 samples were immersed in 1.23% APF gel for 48 hours, a loss of surface glaze and a frosty appearance of the crown could be seen. Group 3.4 samples were immersed in 1.23% APF gel for 72 hours; on comparison with the control sample, a loss of glaze and a chalky appearance of the crown were observed.

Topographical analysis by FE-SEM

**Figure 3:**
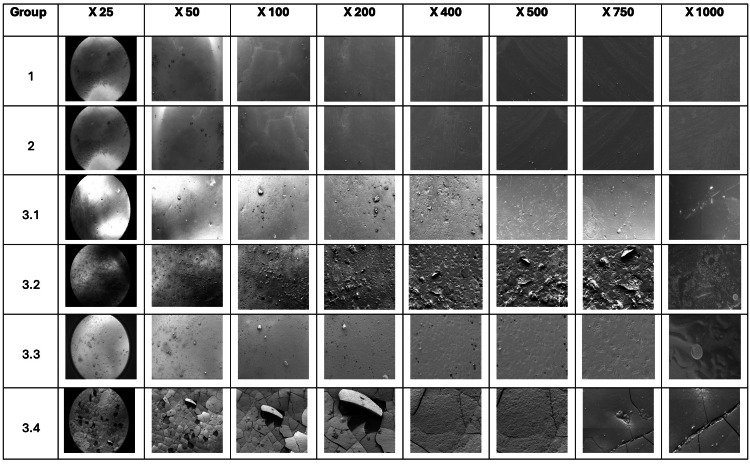
Images showing topographical changes on the surface of zirconia crowns from each group, analysed by FE-SEM at different magnifications. Group 1 samples not immersed in any medium used as a control. Group 2 samples immersed in deionised water. Group 3.1 samples immersed in 1.23% APF gel for four minutes. Group 3.2 samples immersed in 1.23% APF gel for 24 hours. Group 3.3 samples immersed in 1.23% APF gel for 48 hours. Group 3.4 samples immersed in 1.23% APF gel for 72 hours. Image credit: Dr. Sanidhya Makwana. APF, acidulated phosphate fluoride; FE-SEM, field emission scanning electron microscope

The FE-SEM analysis of all groups is shown in Figure 3.

FE-SEM analysis showed a smooth, uniform, continuous, undisrupted and undistorted surface for the control sample (group 1). FE-SEM analysis for the surface texture of zirconia crowns immersed in deionised (distilled) water (group 2) was similar to that of the control (group 1) where the top layer remained intact without any disruption or distortion.

FE-SEM analysis for the surface of zirconia crowns immersed in 1.23% APF gel for four minutes (group 3.1) showed surface scratches and abrasions along with disrupted zirconia dust attached to the surface with very few pinhole porosities. FE-SEM analysis for the surface of zirconia crowns immersed in 1.23% APF gel for 24 hours (group 3.2) showed surface abrasions along with disrupted zirconia dust in larger quantities attached to the surface. Pinhole porosities were seen. FE-SEM analysis for the surface of zirconia crowns immersed in 1.23% APF gel for 48 hours (group 3.3) showed surface etching, as well as a large number of pinhole porosities. There was reduced zirconia dust but a greater number of pinhole porosities. Surface disruption was also evidently seen due to the erosion effect. The FE-SEM analysis of the samples revealed flaws, imperfections and uneven superficial layers with immersion in 1.23% APF gel for 72 hours (group 3.4). It had been observed that surface grains were disrupted and micropores had formed. Material from the top layer had chipped off, and micro-cracks were seen all over the exposed surface.

No change in the mass of the samples was observed when the samples were weighed on a microscale before and after immersion. Thus, net mass loss was zero. This study showed that there was no loss in the volume of zirconia on exposure to deionised water and 1.23% APF gel.

## Discussion

Zirconia has superior mechanical properties as compared to other restorative materials used as fixed dental prostheses [30]. However, there is limited knowledge about the chemical interaction of zirconia with oral fluids and various other oral hygiene agents or food and beverages.

Polished and glazed zirconia showed greater surface roughness post immersion in orange juice and cola, when measured with a profilometer [31]. Beverages such as coffee, cola and protein shake can affect the colour stability of zirconia by staining the surface. Moreover, topical mouthwash agents such as chlorhexidine can alter the surface colour of zirconia as well [32]. Toothpaste and denitrifying agents greatly affected the surface gloss and surface roughness of both polished and glazed zirconia. Toothpaste reduced the surface gloss, but it was within the threshold limits. Denitrifying agents made the surface rough. No change in the translucency of the material was observed [33].

The immersion of zirconia crowns in 1.23% APF gel, which is a commonly applied topical fluoride agent in school-aged children, teenagers and adults with increased risk of root caries [34], was conducted to examine the impact of an acidic electrochemical environment on the outermost layer of zirconia. It was hypothesised that the topical agent’s acidic aqueous environment would degrade the zirconia surface and accelerate the ageing process, resulting in micro-crack nucleation and propagation.

In the present study, the effect of 1.23% APF gel on the zirconia crown was evaluated with FE-SEM. There was a significant change observed on the surface of zirconia crowns immersed in 1.23% APF gel. Mass loss analysis showed no change in the mass of the samples post immersion. It was found that fluoride and acidic pH altered the surface morphology of zirconia crowns. In this study, following the immersion of zirconia crowns in 1.23% APF gel, visual changes on the surface can be observed in Figure 2. There was no visual change on the surface of crowns immersed in deionised water. A marked loss of glaze in visual appearance on the surface of zirconia crowns immersed in 1.23% APF gel for four minutes was observed. The total loss of glaze could be seen on the surface of zirconia crowns immersed in 1.23% APF gel for 24 hours. For the zirconia crowns immersed in 1.23% APF gel for 48 hours, the total loss of glaze and frosty appearance could be seen, while for zirconia crowns immersed for 72 hours in 1.23% APF gel, the total loss of glaze and dull and chalky appearance of the crowns were seen.

On the topographical analysis of the samples with FE-SEM (Figure 3), group 2 samples immersed in deionised water for 72 hours showed similar surface morphology as compared to the control (group 1) where the top layer remained intact without any disruption or distortion.

The FE-SEM analysis of the samples immersed in 1.23% APF gel for four minutes (group 3.1) showed a scratched/abraded top layer. Those immersed for 24 hours (group 3.2) displayed pinhole porosities and abraded top layer of zirconia crowns, whereas samples immersed for 48 hours (group 3.3) showed a greater number of pinhole porosities and largely etched areas. The samples immersed in 1.23% APF gel for 72 hours (group 3.4) showed that surface grains were disrupted along with the formation of micro-cracks. It could be hypothesised that micro-porosities on greater exposure time coalesced to form micro-cracks that eventually chipped away the top layer from the underlying bulk of zirconia.

The results of this study were comparable to those investigated by Thomas et al. [29], where zirconia rods when immersed in deionised water showed no change, but there were topographical changes visible on the SEM analysis of zirconia rods immersed in 1.23% APF gel for varying time periods. The loss of surface continuity and surface disruption was seen in SEM analysis. FE-SEM analysis as compared to SEM analysis provides clearer imaging with reduced distortion. It can reproduce the spatial resolution to 1.5 nm, which is six times the detailed imaging of the sample as compared to conventional SEM. Thus, FE-SEM analysis provided us with the most accurate topographical changes on the surface of the zirconia crown samples following immersion.

Mass loss analysis showed no net loss of the material, which is similar to the result derived in the study by Thomas et al. [29].

Limitations of the study

The static environment of acidic and neutral pH was considered for the study; on the contrary, oral cavity has a dynamic ecology comprising of neutral, acidic or basic pH depending on the general physiology of the body and food ingested. All the conclusions drawn in this study were based on laboratory conditions. In vivo studies are required to conclude our results clinically. Direct visual analysis without any aid was carried out to evaluate the changes in surface morphology, so operator bias could arise as no standard guidelines or scale is available for visual analysis of dental materials.

Future scope of the study

Atomic force microscopy (AFM) analysis can be done to evaluate the changes in surface morphology on the vertical plane. A similar kind of study can be performed with the dynamic exposure of different elements leading to the combination of acidic, neutral and basic pH environment along with intermittent cyclic loading that can simulate the mechanical loading of the crowns as in function. This can help us to know and understand the combined effect of this complex ecosystem on the properties and the longevity of zirconia crowns in vivo.

## Conclusions

Within the testing conditions and limitations, it was concluded that an acidic electrochemical environment can degrade the material properties of zirconia. On the immersion of zirconia crowns in an aqueous acidic medium of 1.23% APF gel, the crowns showed flaws, imperfections and uneven superficial layers. It was observed that surface grains were disrupted and micropores were formed. Visual alternations on the surface of the zirconia crowns could be seen ranging from loss of glaze to chalky white appearance.
